# Infarcted rat myocardium: Data from biaxial tensile and uniaxial compressive testing and analysis of collagen fibre orientation

**DOI:** 10.1016/j.dib.2016.08.005

**Published:** 2016-08-04

**Authors:** Mazin S. Sirry, J. Ryan Butler, Sourav S. Patnaik, Bryn Brazile, Robbin Bertucci, Andrew Claude, Ron McLaughlin, Neil H. Davies, Jun Liao, Thomas Franz

**Affiliations:** a Division of Biomedical Engineering, Department of Human Biology, University of Cape Town, Observatory 7925, South Africa; b Department of Biomedical Engineering, University of Medical Sciences and Technology, P.O. Box 12810, Khartoum, Sudan; c Department of Clinical Sciences, College of Veterinary Medicine, Mississippi State University, Mississippi, MS 39762, USA; d Tissue Bioengineering Laboratory, Department of Agricultural and Biological Engineering, Mississippi State University, Mississippi, MS 39762, USA; e Cardiovascular Research Unit, Chris Barnard Division of Cardiothoracic Surgery, University of Cape Town, Observatory 7925, South Africa; f Centre for High Performance Computing, Rosebank 7700, South Africa; g Bioengineering Science Research Group, Engineering Sciences, Faculty of Engineering and the Environment, University of Southampton, Southampton SO171BJ, UK

## Abstract

Myocardial infarction was experimentally induced in rat hearts and harvested immediately, 7, 14 and 28 days after the infarction induction. Anterior wall infarct samples underwent biaxial tensile and uniaxial compressive testing. Orientation of collagen fibres was analysed following mechanical testing. In this paper, we present the tensile and compressive stress–strain raw data, the calculated tensile and compressive moduli and the measured angles of collagen orientation. The presented data is associated with the research article titled “Characterisation of the mechanical properties of infarcted myocardium in the rat under biaxial tension and uniaxial compression” (Sirry et al., 2016) [1].

**Specifications Table**

**Table:** 

Subject area	*Mechanics, Biology*
More specific subject area	*Biomechanics of myocardial infarction*
Type of data	*Graphs, tables*
How data was acquired	*Biaxial tensile test, uniaxial compressive test, histological analysis*
Data format	*Raw, analysed*
Experimental factors	*Mechanical testing was performed with tissue samples completely submerged in* phosphate buffered saline at *37* *°C.*
Experimental features	*60:60 N/m biaxial load, 30% wall compression, collagen orientation at 10 transmural sections*
Data source location	*Mississippi State University, Mississippi, USA and University of Cape Town, Cape Town, South Africa*
Data accessibility	*All data is provided in this article*

**Value of the data**

• Full presentation of biaxial mechanical data and collagen fibre orientation for healing myocardial infarcts.
• The presented data demonstrates the mechanical and structural anisotropy of the healing myocardial infarcts.
• First report to characterize the compressive properties of the healing rat myocardial infarcts.
• Presentation of collagen fibre orientation in healing myocardial infarcts.
• The presented mechanical and structural data can be utilised in constitutive modelling of healing myocardial infarcts for rats.

## 1 Data

**Fig. 1:**
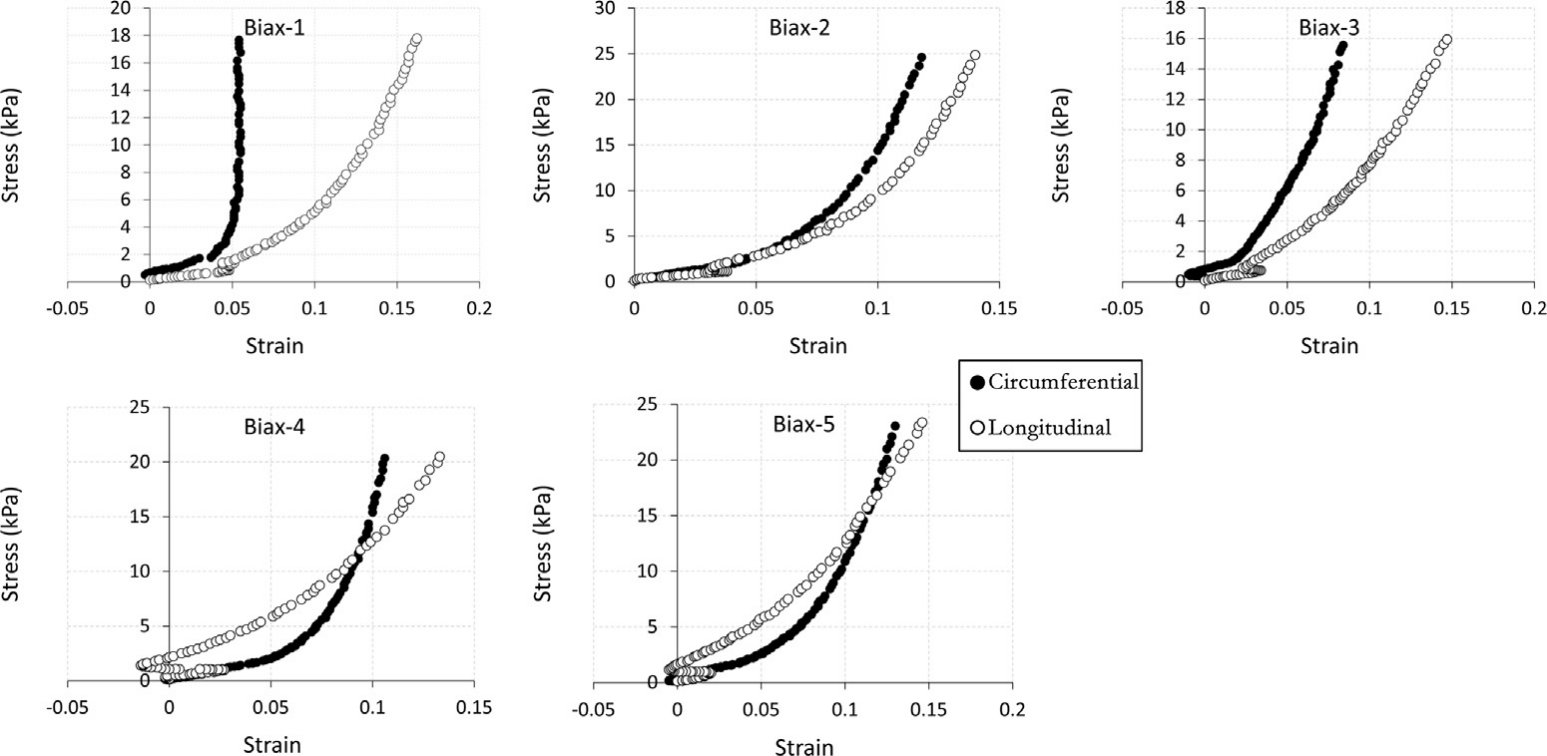
60:60 N/m biaxial tensile stress–strain relationship for individual samples (*n*=5) from 0 day infarct group. Biaxial test raw data are provided in Supplementary Data Set A.

**Fig. 2:**
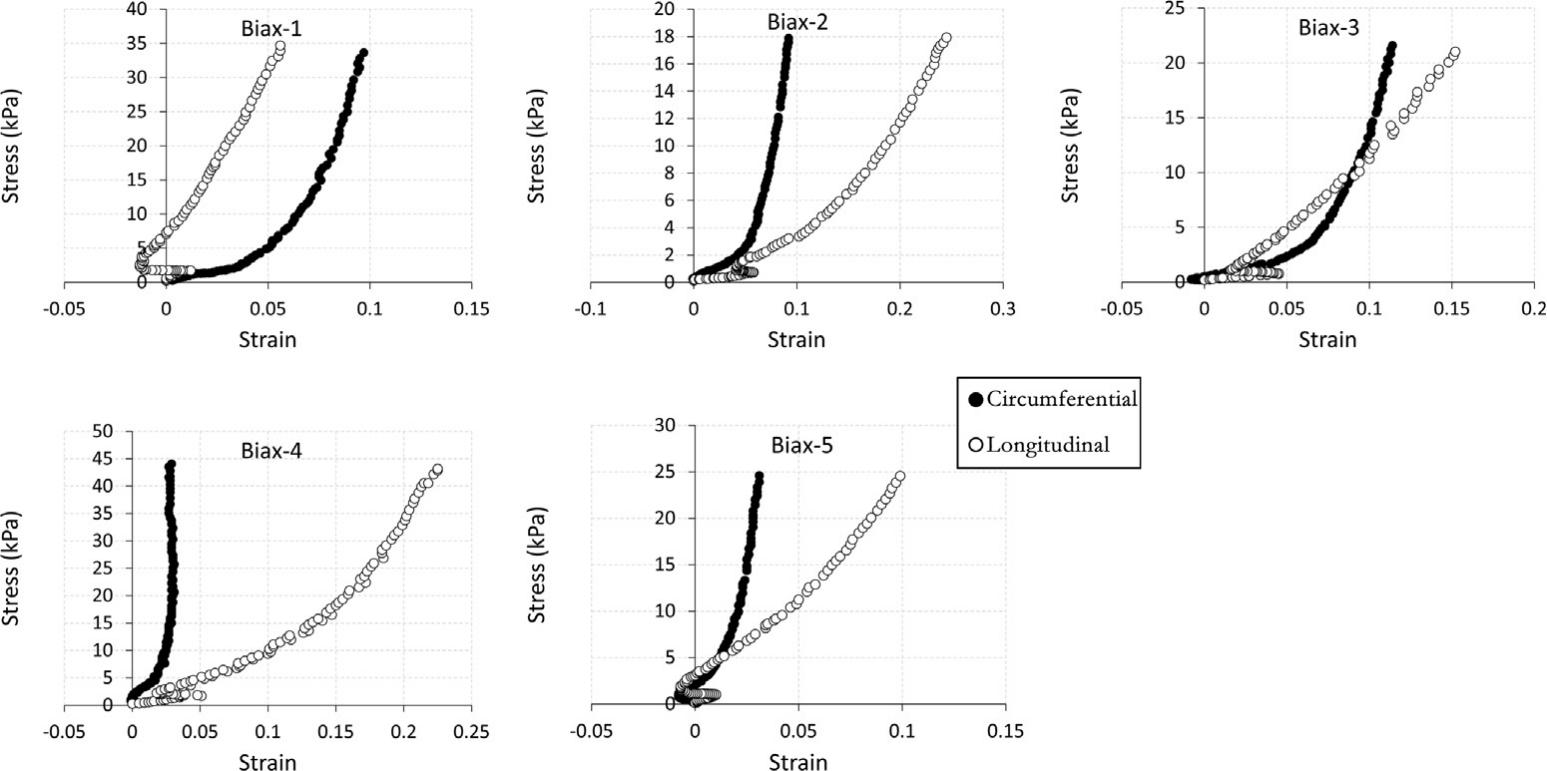
60:60 N/m biaxial tensile stress–strain relationship for individual samples (*n*=5) from 7 day infarct group. Biaxial test raw data are provided in Supplementary Data Set A.

**Fig. 3:**
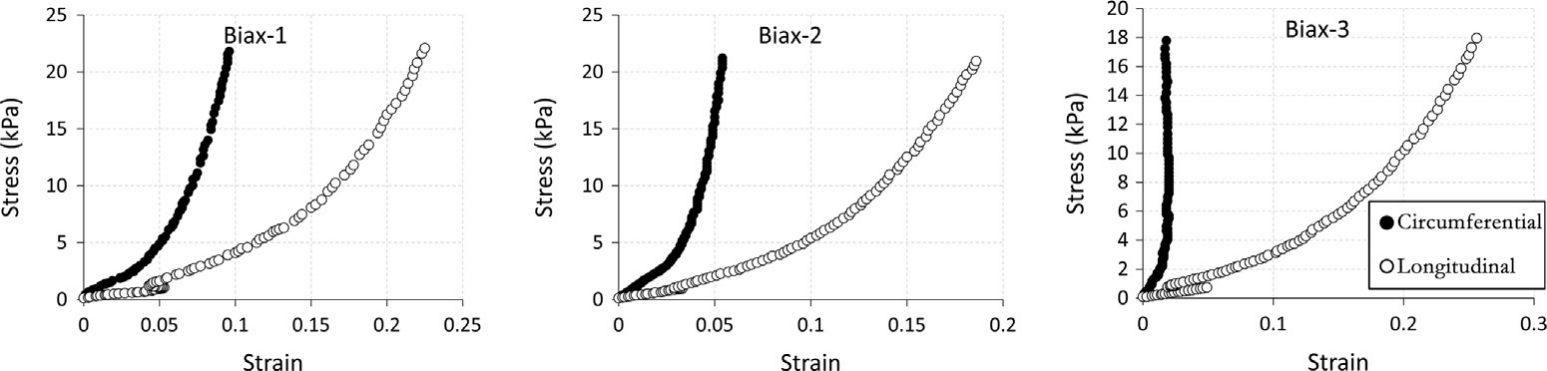
60:60 N/m biaxial tensile stress–strain relationship for individual samples (*n*=3) from 14 day infarct group. Biaxial test raw data are provided in Supplementary Data Set A.

**Fig. 4:**
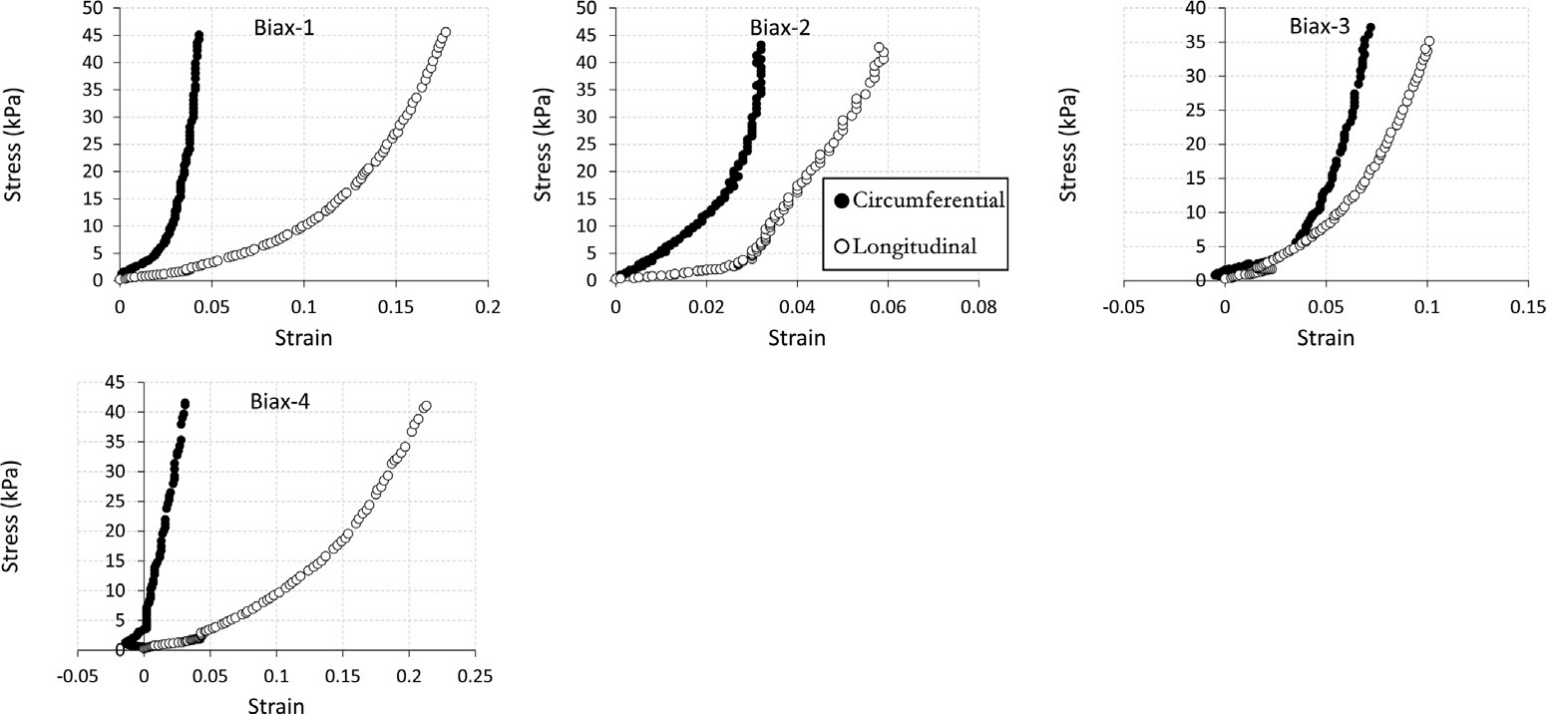
60:60 N/m biaxial tensile stress–strain relationship for individual samples (*n*=4) from 28 day infarct group. Biaxial test raw data are provided in Supplementary Data Set A.

**Fig. 5:**
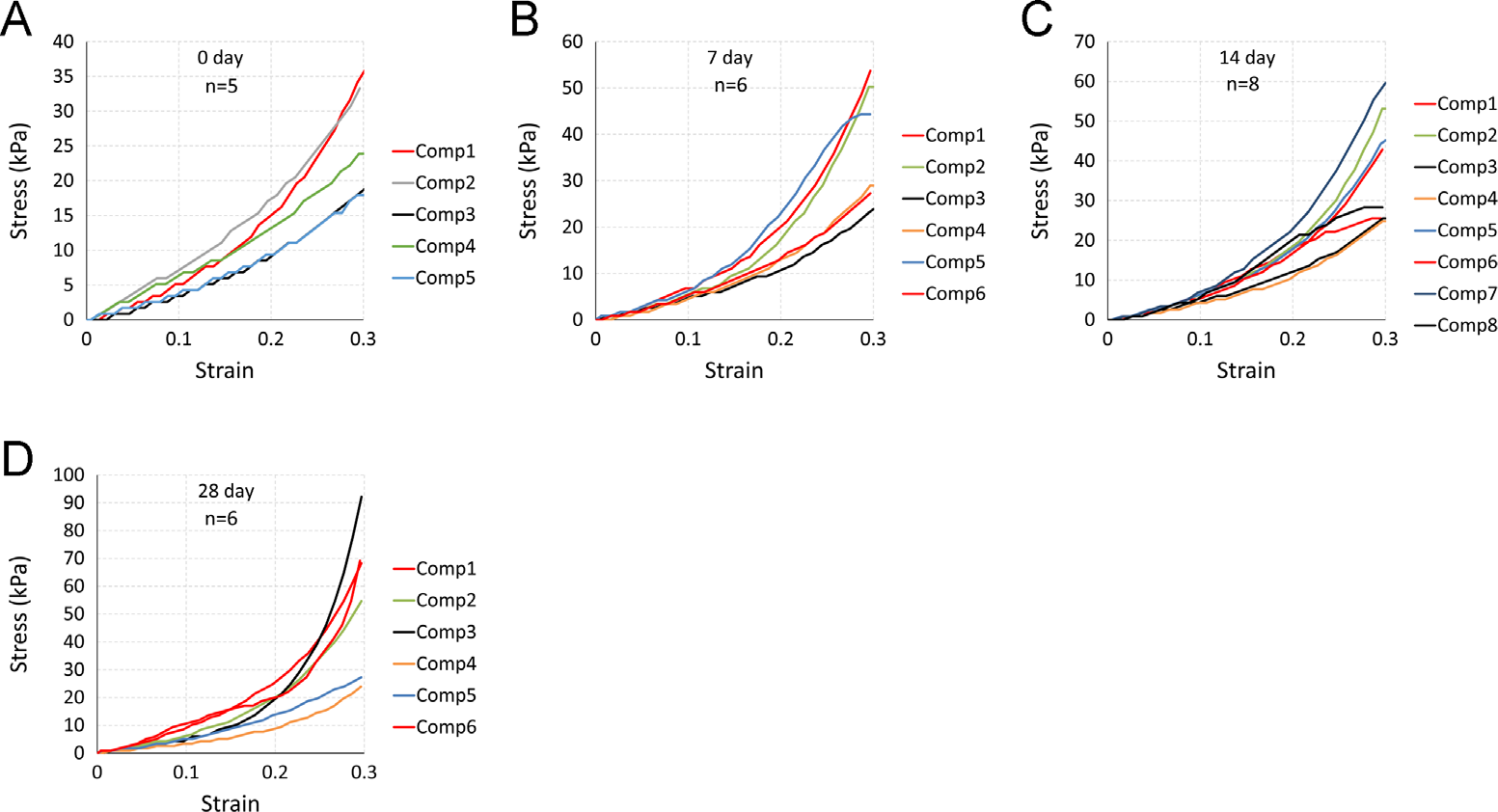
Compressive stress–strain relationship for individual samples of 0 (a), 7 (b), 14 (c) and 28 day and (d) infarct groups. Compression test raw data are provided in Supplementary Data Set B.

The data being shared describe the mechanical and structural properties of healing rat myocardial infarcts from individual samples in several post-infarct time points: immediately (i.e. 0 day), 7, 14 and 28 days after the induction of myocardial infarction. The mechanical data are composed of biaxial tensile stress–strain relationships (Fig. 1, Fig. 2, Fig. 3, Fig. 4), biaxial tensile moduli (Table 1), compressive stress–strain relationships (Fig. 5) and compressive moduli (Table 2). The structural data include the angles of collagen fibres orientation measured at the centre of infarct samples (Table 3).

## 2 Experimental design, materials and methods

The details of the experimental work and materials are provided in [1].

## Figures and Tables

**Table 1:** Circumferential and longitudinal tensile moduli (kPa) calculated for individual samples of different infarct groups.

	Circumferential				Longitudinal			
	0 day (kPa, *n*=5)	7 day (kPa, *n*=5)	14 day (kPa, *n*=3)	28 day (kPa, *n*=4)	0 day (kPa, *n*=5)	7 day (kPa, *n*=5)	14 day (kPa, *n*=3)	28 day (kPa, *n*=4)
	534	644	545	1522	227	466	240	341
	294	494	1168	1689	380	106	243	1105
	571	416	1432	714	177	136	153	337
	373	1125		947	187	315		166
	419	787			267	228		
Mean±SD	438±114	693±280	1048±455	1218±462	248±82	250±146	212±51	487±420

**Table 2:** Compressive moduli (kPa) calculated for individual samples of different infarct groups.

	0 day (kPa, *n*=5)	7 day (kPa, *n*=6)	14 day (kPa, *n*=8)	28 day (kPa, *n*=6)
	244	429	73	567
	188	388	460	430
	99	148	162	1060
	109	192	160	188
	87	154	397	155
		172	343	732
			415	
			72	
Mean±SD	145±68	247±126	260±160	522±344

**Table 3:** Collagen orientation angles (deg) at 10 transmural sections from samples of 7, 14 and 28 day infarct groups. Collagen orientation raw data are provided in Supplementary Data Set C.

		Epi-	Transmural section (depth)								Endo-
		S1	S2	S3	S4	S5	S6	S7	S8	S9	S10
7 day	MA (deg)	−31.0	−31.1	−43.1	−39.9	−30.7	−30.9	−33.8	−36.0	−20.3	−27.9
	MVL	0.75	0.75	0.94	0.90	0.84	0.86	0.89	0.91	0.82	0.84
	CSD (deg)	43.1	43.7	19.8	25.8	34.0	31.6	27.8	24.4	35.9	34.0
14 day	MA (deg)	−33.8	−21.5	−18.8	32.3	−8.3	32.4	−20.9	−25.5	−27.4	−29.8
	MVL	0.79	0.75	0.77	0.83	0.69	0.87	0.83	0.76	0.78	0.85
	CSD (deg)	39.9	43.9	41.5	35.0	49.3	29.9	35.2	42.7	40.2	32.3
28 day	MA (deg)	−6.4	1.3	−15.4	−13.9	−35.7	−34.3	−33.2	−35.3	0.4	−10.2
	MVL	0.76	0.66	0.79	0.79	0.77	0.80	0.78	0.79	0.70	0.71
	CSD (deg)	42.2	52.1	39.8	39.0	41.5	38.3	40.6	39.2	48.7	47.0

MA=mean angle; MVL=mean vector length; CSD=circular standard deviation.

## Footnotes

Appendix A Supplementary data associated with this article can be found in the online version at doi:10.1016/j.dib.2016.08.005.
