# Supplementary material for: Infarcted rat myocardium: Data from biaxial tensile and uniaxial compressive testing and analysis of collagen fibre orientation
Source: Data Brief. 2016 Aug 4;8:1338–43. doi: 10.1016/j.dib.2016.08.005 (PMC4992040; doi:10.1016/j.dib.2016.08.005)
Supplement: Supplementary file 2 — Supplementary material [file mmc2.zip › Supp.zip!/Data in Brief/~WRL1489.tmp]

**Instructions when you submit your Data in Brief with your research article**

1. Complete this template below. Make sure you reference all data files directly in this template at the appropriate point.
2. Zip this completed Word document and any data files relevant to the Data in Brief (whichever supplementary data files you have chosen to include) into a single. Zip file. When you submit your revised research article, please upload this .zip file as a “Data in Brief” item.
3. Double check in your research article that any reference to supplementary files that have been converted to your Data in Brief article now properly reference the Data in Brief instead. i.e. (see supplementary Figure 1) should now say, (see Figure 1 in Ref [#]) where your Data in Brief article is included in the reference list of your research article (including title, authors, journal name *Data in Brief* and the text “*submitted”*)
4. Make sure you reference your associated research article in the reference list here as well. You may reference this as “in press”

GENERAL INFORMATION

Throughout your entire Data in Brief (DiB) article keep in mind that you are simply describing data and not providing conclusions/interpretive insights. Please avoid using words such as 'study, 'studied, 'results', 'conclusions', etc. Please do use the word “data” throughout your DiB paper wherever possible.

*[please fill in this template below and delete all instruction text above and below before submitting]*

*Data article*

**Title:** *Mechanical properties and structure of infarcted myocardium in the rat under biaxial tension and uniaxial compression*

**Authors:** Mazin S. Sirry^1,2^, J. Ryan Butler^3^, Sourav S. Patnaik^4^, Bryn Brazile^4^, Robbin Bertucci^4^,

Andrew Claude^3^, Ron McLaughlin^3^, Neil H. Davies^5^, Jun Liao^4^, Thomas Franz^1,6,7,8,*^

**Affiliations:** ^1^Division of Biomedical Engineering, Department of Human Biology, University of Cape Town, Observatory 7925, South Africa

^2^Department of Biomedical Engineering, University of Medical Sciences and Technology, P.O. Box 12810, Khartoum, Sudan

^3^Department of Clinical Sciences, College of Veterinary Medicine, Mississippi State University, Mississippi, MS 39762, USA

^4^Tissue Bioengineering Laboratory, Department of Agricultural and Biological Engineering, Mississippi State University, Mississippi, MS 39762, USA

^5^Cardiovascular Research Unit, Chris Barnard Division of Cardiothoracic Surgery,
University of Cape Town, Observatory 7925, South Africa

^6^Centre for High Performance Computing, Rosebank 7700, South Africa

^7^Bioengineering Science Research Group, Engineering Sciences, Faculty of Engineering and the Environment, University of Southampton, Southampton SO171BJ, UK

**Contact email:** [**thomas.franz@uct.ac.za**](mailto:thomas.franz@uct.ac.za)

**Abstract**

*[Explicitly tell the reader what data and information they will find in this data article. Please only describe the data contents presented in this DiB article and do not describe your related research article. The DiB abstract should be purely descriptive (i.e., no results, conclusions or insightful observations about the data) If the data you describe in this article is hosted in a public repository instead of directly with this data article, state the repository name and reference number. You may also directly point the reader to your research article for further interpretation and discussion, here in the abstract]*

**Specifications Table** *[please fill in right-hand column of the table below]*

| Subject area | *Physics, Chemistry, Biology, Economics, Psychology* |
| --- | --- |
| More specific subject area | *Describe narrower subject area* |
| Type of data | *Table, image (x-ray, microscopy, etc), text file, graph, figure* |
| How data was acquired | *Microscope, survey, SEM, NMR, mass spectroscopy, etc. If an instrument was used, please provide the model and make of the instrument* |
| Data format | *Raw, filtered, analyzed, etc* |
| Experimental factors | *Brief description of any pretreatment of samples* |
| Experimental features | *Very brief experimental description* |
| Data source location | *City, Country and/or Latitude & Longitude (& GPS coordinates) for collected samples/data if applicable* |
| Data accessibility | *State if data is with this article or in public repository. If public repository, please explicitly name repository and data identification number and provide a direct URL to data* |

**Value of the data** *[Describe in 3-5 bulleted points why this data is of value to the scientific community.* *Broadly explain to other researchers how the data could be potentially valuable to them, with an eye towards possibly opening up doors for new collaborations. For example, how could this data: be compared to other data for further insight, serve as a benchmark for other researchers, be used in the development of further experiments in a particular area, etc. Please do not offer interpretative statements or conclusions about the data, nor state why this data was valuable for an already-published research study.]*

**Data**

[*Briefly describe the data you are sharing with this data article here, to give the reader context before you describe the materials and methods]*

1. Individual equibiaxial stress-strain curves (provide raw data as online supplement)
2. Circumferential and longitudinal tensile elastic modulus for individual samples (table in paper, data as online supplement)
3. Compressive stress-strain curves (data as online supplement)
4. Cross-sectional area of tissue sections and infarcts
5. Collagen fibre orientation

Figure 1: 0 day infarct samples (n=5)

Figure 2: 7 day infarct samples (n=5)

Figure 3: 14 day infarct samples (n=3)

Figure 4: 28 day infarct samples (n=4)

Figure 5: Compression

**Experimental Design, Materials and Methods**

[*Complete description of the Experimental design and methods used to acquire the data and where applicable, in the analysis. Include any relevant figures/tables needed to fully understand the data. Please also provide, where applicable, any code files used to provide base-level analysis or filtering of the data.*]

*[(NO) Conclusions/Summary: DiB papers are distinctly different from research articles and should* **not** *include interpretations and conclusions. Do not include a Conclusion or Summary section.]*

**Acknowledgements**

**References** *[please include all references relevant to the data described here; references are not limited]*
